# Unexpected diversification of DENV2 genotype III in Colombia: New Insights and application of the globalized nomenclature

**DOI:** 10.1371/journal.pone.0343528

**Published:** 2026-02-24

**Authors:** Eliana P. Calvo, Leidy Johana Madroñero, Lenny Marcela Hernández, Jhann Andrés Arturo, Hernando Pinzón, Félix Giovanni Delgado, Myriam Lucia Velandia-Romero, Verity Hill, Nathan D. Grubaugh, Jaime Eduardo Castellanos

**Affiliations:** 1 Grupo de Virología, Vicerrectoría de Investigaciones, Universidad El Bosque, Bogotá, Colombia; 2 Fundación Hospital Infantil Napoleón Franco Pareja - Casa del Niño, Cartagena, Colombia; 3 Department of Epidemiology of Microbial Diseases, Yale School of Public Health, New Haven, Connecticut, United States of America; Universidad Cooperativa de Colombia, COLOMBIA

## Abstract

Dengue is the most prevalent mosquito-borne viral disease worldwide and constitutes a major public health concern in Colombia. The disease is caused by four antigenically distinct dengue virus serotypes (DENV-1 to DENV-4), which share more than 65% genome identity. Although all four serotypes co-circulate in Colombia, secondary infections with DENV-2 have frequently been associated with more severe clinical outcomes. DENV-2 comprises six genotypes, of which genotype III has been the dominant lineage in the Americas over the past two decades. In this study, we investigated the evolutionary dynamics of DENV-2 genotype III in Colombia by analyzing available whole-genome coding sequences together with twenty-six newly generated genomes. Bayesian and maximum-likelihood phylogenetic approaches were applied to infer evolutionary relationships and temporal patterns. To harmonize lineage definitions and facilitate regional comparisons, we adopted the recently proposed hierarchical lineage nomenclature and used the Genome Detective Dengue typing tool for automated classification and consolidation of sequences from Colombia, Ecuador, and Venezuela. Phylogenetic analyses revealed a broadly distributed major lineage (previously designated D) circulating across the three countries, together with ongoing diversification and repeated introductions of genotype III within Colombia. Four minor lineages were identified, designated D.2.1, D.2.2, D.3.1, and D.3.2. Notably, the diversification of these minor lineages was accompanied by multiple non-synonymous substitutions. In particular, the two lineages currently circulating in Colombia, D.2.1 and D.2.2, are distinguished by approximately 20 mutations; however, the functional implications of these substitutions for viral virulence, pathogenicity, or vector competence remain unknown. These findings support the need for sustained and targeted genomic surveillance to detect and monitor emerging dengue virus lineages, prioritize them for epidemiological follow-up, and inform geographically focused public-health strategies. Overall, this study highlights the value of a globalized nomenclature, which enables the integration of genomic data across countries, facilitates the identification of emerging lineages in the region, and supports molecular surveillance efforts aimed at assessing their potential impact on disease presentation and public health.

## Introduction

Dengue is a mosquito-borne disease caused by the dengue virus (DENV) and transmitted primarily by *Aedes aegypti* and *A. albopictus* mosquitoes. These vectors are distributed throughout tropical and subtropical regions worldwide. With an estimated 390 million infections per year [1] and nearly half of the world’s population at risk, dengue represents the greatest human disease burden of any arbovirus [2]. Over the past two decades the reported global incidence has increased dramatically, with large outbreaks and substantial mortality in recent years, particularly in the Americas. In 2024 the Region of the Americas reported a marked surge in dengue cases and deaths, underscoring the ongoing public-health challenge [2]. Despite the implementation of established prevention measures—including vector control, environmental management, community engagement and clinical management—the sustained increase in dengue incidence suggests that these interventions have not been sufficient to curb transmission in many endemic settings [3].

DENV infection produces a wide clinical spectrum. Approximately 75% of infections are asymptomatic; among symptomatic individuals most develop a mild, self-limited febrile illness known as dengue fever (DF). Around 5% of infections progress to severe dengue, formerly dengue hemorrhagic fever (DHF), which can be life-threatening. Severe disease is associated with endothelial dysfunction and plasma leakage, and may present with ascites, pleural effusion, hypovolemic shock, hepatic impairment and hypotension, often accompanied by bleeding manifestations [4].

DENV is a single-stranded, positive-sense RNA virus of the family *Flaviviridae* (genus *Orthoflavivirus*) with an ≈ 11 kb genome that encodes three structural proteins (C, prM/M and E) and seven non-structural proteins (NS1–NS5). Four antigenically distinct serotypes (DENV-1 to DENV-4) co-circulate globally, sharing roughly 65–70% genome identity. Within each serotype multiple genotypes have been described—often reflecting historical geographic patterns—typically differing by ~6–8% at the nucleotide level and ~3% at the amino-acid level. [5]. Importantly, genotype identity and geographic distribution are not merely descriptive. Distinct genotypes can differ in replicative fitness, vector competence, and transmission potential [6,7]. Genotypic variation may also alter antigenic properties and neutralization sensitivity, with potential implications for vaccine performance [8,9].

Historically, all four DENV serotypes have circulated in the Americas since the early 20th century, primarily causing DF cases. However, the first major epidemic of DHF caused by DENV-2 in the region, occurred in 1981 in Cuba and spread through the Caribbean and South America by the late 1980s [10]. This epidemic was linked to the introduction of the Asian–American (AS/AM) genotype which subsequently replaced the native American genotype [11]. In Colombia, the AS/AM genotype remained the dominant for decades until the Cosmopolitan genotype was introduced in 2023 [12].

In Colombia, although several studies have analyzed the evolutionary history and spatiotemporal dynamics of DENV-2 [13–15], most have been conducted in specific geographical regions and focused on sequences of the envelope protein (E) coding region (‘gene’). This approach potentially overlooks valuable information that could be provided by the evolution of non-structural proteins. Therefore, this study proposes to analyze the spatiotemporal dynamics of genotype III using all complete genomes reported in the GISAID database for Colombia, as well as new viral sequences obtained from the Bolivar province in late 2021.

## Materials and methods

### Sample collection and virus detection

This retrospective study utilized 26 human plasma samples collected from various localities across Colombia, representing the circulating viruses over the past decade (Table 1). Viruses were isolated by culturing in C6/36 mosquito cells from patient sera collected in previous studies [16,17]. The study also included anonymous samples of residual blood samples collected for laboratory diagnosis at the Fundación Hospital Infantil Napoleón Franco Pareja, from 1 July to 30 December 2021. Samples were not collected prospectively or specifically for this project. The samples were anonymized using a code before being sent to Universidad El Bosque and no personal identifiers or clinical data of the patients were obtained with the samples. The study was approved by the Research and Ethics Committee of the Hospital Infantil Napoleón Franco Pareja on May 3, 2019. The Ethics Committee granted a waiver of informed consent because the research used anonymized retrospective samples and posed minimal risk.

**Table 1:** Summary of sequences used in this study.

Country	Maximum Likelihood dataset	Molecular Clock subset	Sampling Dates	Lineage assigned by Genome Detective tool
Bolivia	3	–	2018-2022	C
Brazil	195	23	1995-2024	B
				C
				C.1.1
Cambodia	2	–	2001-2002	A
Cameroon	1	–	2014	C
**Colombia**	**135**	**59**	**1998-2024**	**B**
				**D.2**
				**D.3**
Cuba	1	–	2019	C.2
Dominican-Rep	6	–	2023	C.1.2
Ecuador	139	21	2014-2024	D.2
				D.3
El-Salvador	6	1	2019	D.1
Guatemala	8	3	2010-2024	D.1
Guyana	1	1	2000	B
Honduras	3	2	2023-2024	D.1
Martinique	1	–	2005	D.1
Mexico	5	4	2007-2021	D.1
Nicaragua	11	5	2005-2018	D.1
Panama	1	–	2011	D.1
Papua NG	1	–	2003	A
Reunion	1	–	2014	C
Peru	10	10	2002-2018	B
				C
Puerto-Rico	48	15	1989-2023	B
				C.1.2
				D.1
USA	74	–	2010-2024	C.1.2
				C.2
				D.1
Venezuela	34	18	1990-2016	B
				D.2
				D.3
Vietnam	12	–	1987-2006	A
TOTAL	698	161		

Viral RNA was extracted using the RTP® Pathogen Kit (Invitek, Germany) following the manufacturer’s instructions. DENV detection was performed by reverse transcription-quantitative polymerase chain reaction (RT-qPCR) using specific primers and probes [18]. DENV2-positive samples with Ct values <26 were selected for library preparation and whole genome sequencing.

### Virus isolation

Samples were diluted 1:20 and inoculated onto C6/36 cells grown in RPMI medium supplemented with 2% fetal bovine serum (FBS). After 10 days of culture at 33°C and 5% CO_2_, the supernatant was recovered by centrifugation, supplemented with 10% FBS, and stored at −80°C until use. DENV confirmation was performed by RT-qPCR as described above. Positive samples with Ct values >26 were passed once more on C6/36 cells, and supernatants were harvested after 7 days of inoculation for DENV detection.

### Full genome amplification and sequencing

Reverse transcription was carried out using 10 μL of RNA, 4 μL LunaScript® RT SuperMix 5X (NEB #E3010), which contains murine RNase inhibitor, random hexamer and poly-dT primers. The reaction was performed in a T100 BioRad thermal cycler under the following conditions: 25°C for 5 min, 55°C for 30 min, and 95°C for 1 min. PCR amplification of the complete genome was performed in two independent reactions using 6 μL of cDNA, 10 μL Q5® High-Fidelity 2X Master Mix (NEB), and 4 μL of each primer pool (0.15 nM) as described by Stubbs et al [19] and modified in this study (S1 Table). Amplification conditions were 98°C for 1 min, followed by 40 cycles of 98°C for 30 s and 60°C for 5 min. Amplicons were mixed, diluted 1:10 with nuclease-free water, and quantified by fluorometric assay. A minimum of 50 ng DNA was used for library preparation using NEB reagents and protocols for end repair, barcode ligation, and adaptor ligation. Pooled libraries were purified and sequenced on a R10.4 flow cell MinION (ONT, Oxford Nanopore Technology).

### Genome reconstruction

Sequences were analyzed using a five-step pipeline. First, base calling was performed using *Guppy v6.5.7* (Oxford Nanopore Technologies) to generate FASTQ files from raw signal data (FAST5). Second, demultiplexing and barcode removal were conducted using *Guppy barcoder*, producing individual FASTQ files for each sample. Third, primer trimming was carried out with *Cutadapt v3.5*, using a multi-FASTA file containing the sequences of all primers used in the multiplex PCR (S1 Table). Fourth, read filtering (200–700 nt) and chimera removal were performed with *Guppyplex* to retain high-quality reads. Finally, genome assembly was conducted using *Bowtie2*, with the DENV-2 reference genome (GenBank accession number NC_001474.2).

### Data collection and phylogenetic analysis

All available complete Asian-American DENV2 genomes (>10,000 bp) with associated collection dates and origins, were downloaded from GISAID [20]. The EPI_SET_250213qu comprises 2,041 sequences, collected between 01/01/1987 and 15/01/2025, and is available at gisaid.org https://doi.org/10.55876/gis8.250213qu. To this data set, we added 26 novel Colombian sequences reported here. A total of 2,066 genomes were aligned using MAFFT v7.490 and then, identical and highly similar (>99%) sequences were removed using CD-HIT. The final dataset comprised 698 sequences from 24 countries. Genotypes, major and minor lineages were assigned using Dengue Virus Typing Tool nomenclature system (Genome Detective, https://www.genomedetective.com). A maximum likelihood (ML) phylogenetic tree was inferred using the ultrafast bootstrap approximation (UFBoot) in IQ-TREE software v2.0.7 and 1,000 bootstrap replicates [21].

### Spatiotemporal dispersion pattern analysis

For this analysis, the dataset of 698 sequences was downsized to minimize the over-representation of some sequences; genomes from the same location and collected in the same year were removed, as were sequences without a collection location or containing long stretches of Ns. Thus, the temporal signal was assessed on 161 sequences from the major lineages circulating in the Americas, through a root-to-tip regression analysis performed in TempEst [22]. A comparison between the strict and relaxed molecular clock models was performed using marginal likelihood estimates (MLE) obtained through path sampling (48 steps, chain length = 1,000,000; α = 0.6; burn-in = 10%; pre-burn-in = 100,000). The Bayes Factor (BF) was calculated as: BF = 2 × (MLE_Relaxed_ − MLE_Stric_). According to Kass and Raftery [23] scale, a BF > 10 was considered a decisive support in favor of the relaxed model.

Ancestral reconstruction and discrete phylogeography analyses were performed using the GTR + Γ4 model, a relaxed molecular clock, and a Bayesian skyline coalescent tree prior. Discrete phylogeography with a symmetric model was selected and an effective sample size (ESS > 200) was achieved using 100M generations. Log files were analyzed in Tracer v1.7 [24]. Maximum clade credibility (MCC) trees were summarized using TreeAnnotator v1.8 [25] and visualized with FigTree v1.4.4 [26]. Mean nucleotide substitution rate, time to the most recent common ancestor (TMRCA), geographic origin, and overall spatial dynamics were inferred using Bayesian Markov chain Monte Carlo (MCMC) statistical framework in BEAUti/BEAST v2.7.6 package [27].

### Selection pressure analysis

The Colombian dataset, comprising 59 sequences, underwent selection pressure analysis using maximum-likelihood methods (default p < 0.1) on the Datamonkey web-server (https://www.datamonkey.org/) [28]. These methods included Fixed Effect Likelihoods (FEL) [29], Single Likelihood Ancestor Counting (SLAC) [29], Mixed Effects Model of Evolution (MEME) [30], and Fast Unconstrained Bayesian AppRoximation (FUBAR) for inferring selection [31]. Sites were considered under positive selection if at least three methods showed ω > 1 (ω = nonsynonymous/synonymous substitution ratio). Default significance levels were used (SLAC, FEL, MEME: p-value < 0.1; FUBAR: posterior probability = 0.9).

## Results

In this study, we have adopted the recently proposed system of classification and nomenclature for DENV lineages [32]. This standardized approach to classification enables more precise comparisons across studies and enhances our understanding of DENV evolution and epidemiology. By adhering to this system, we contribute to the broader effort of harmonizing DENV genetic research and surveillance worldwide. Following this nomenclature, we refer to the AS/AM genotype as genotype III, and we use the terms “major lineages” and “minor lineages” to describe what were previously known as clades and subclades, respectively.

### Genome sequencing and genotype assignation

Twenty-six near full-length DENV-2 sequences were obtained and deposited in GenBank database under accession numbers PQ363530–55. One from Huila collected in 2011, five from Cundinamarca (2013–2016), 17 from Bolivar collected during 2021 and three from Vichada. The sequences had an average length of 10,650 nucleotides, indicating the loss of the last 85 nucleotides of the 3’ UTR. Coverage was higher than 90% and depth > 30X for 24 out of the 26 reported sequences (S2 Table). The ORF located between nucleotides 97 and 10,267 was 10,170 nucleotides long, encoding a polyprotein of 3,390 amino acids.

The newly generated sequences, along with 672 available genomes in GISAID database, were evaluated using the Dengue Virus Typing Tool available at Genome detective (https://www.genomedetective.com). The lineage assignment showed four major lineages for the genotype III, two of which are currently circulating in the Americas (Table 1). The typing tool also detected minor lineages, revealing two patterns of distribution: minor lineage D.1 in Central American countries, and lineages D.2 and D.3 in South American countries.

**Fig 1:**
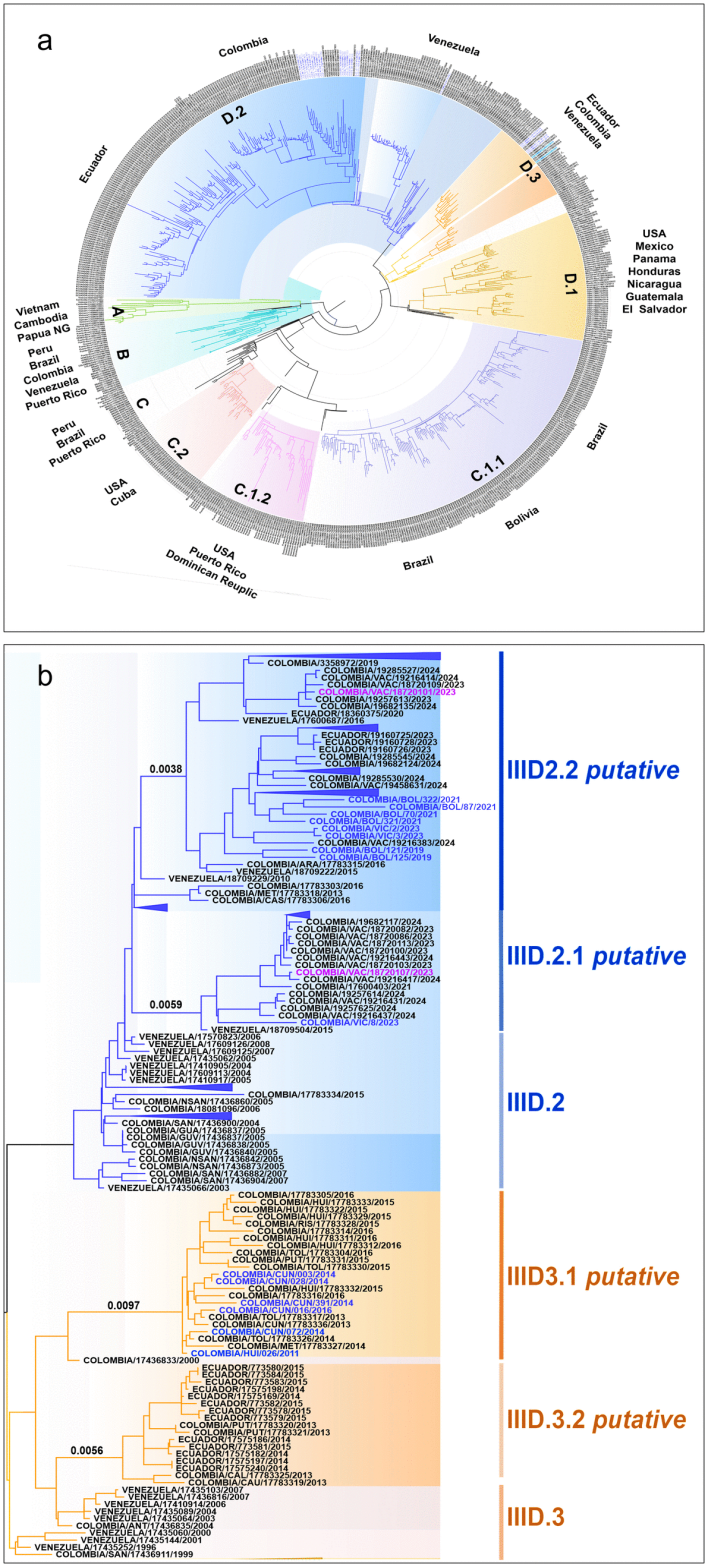
Phylogenetic analysis of the dengue virus serotype 2, genotype III. A Maximum likelihood phylogenetic tree was inferred using IQ-TREE program in ModelFinder. Node support was evaluated using ultrafast bootstrap (UFBoot) and 1,000 replicates. **a.** Full reconstruction of 698 sequences from 1987–2024 colored by lineage assignment. **b** Detail of DENV2III_D major lineage. The tree is drawn to scale, with branch lengths measured in substitutions per site. The number of substitutions were calculated by multiplying the branch length by the genome alignment length (10,170 nucleotides).

Then, we conducted a Maximum likelihood analysis which revealed at least four main clades corresponding to the major lineages A, B, C and D defined by Hill et al. [32] for this genotype (Fig 1). Major lineage A is characterized by strains that circulated in Asia, particularly in Vietnam and Cambodia, between 1980 and 2000. Major lineage B includes sequences that circulated in Puerto Rico, Venezuela, Brazil, Peru and Colombia in the 1990s and early 2000s. Major lineage C comprises at least three subclades, which correspond to the minor lineages designated by Hill et al. as C.1.1, C.1.2 and C.2. The C.1.1 comprises Brazilian and Peruvian sequences, while C.1.2 comprises sequences from the United States, the Dominican Republic and Puerto Rico. As for lineage D, minor lineage D.1 groups sequences from Central America (Mexico, El Salvador, Nicaragua, and Guatemala), while D.2 and D.3 bring together sequences circulating in Colombia, Venezuela, and Ecuador. These findings suggest a geographical diversification of the lineages (Fig 1).

### Detection of new minor lineages in Colombia

Given that the two predominant minor lineages in Colombia (D.2 and D.3) are separated into distinct subclades (Fig 1a), we evaluated whether they could be considered new minor lineages, based on the criteria set out by Hill et al. [32] for establishing minor lineages: the presence of a cluster of at least 15 sequences, 20 nucleotide substitutions inferred along the ancestral branch, and a sister branch at the same level.

For the minor lineage D.3, the phylogenetic tree revealed the divergence of two sister subclades. The first, grouped Colombian sequences and it was separated from the common ancestor by 0.0097 substitutions per site, corresponding to 98 inferred substitutions. As all three requirements were met, the subclade was designated as D.3.1 *putative* (Fig 1b). This minor lineage appears to be unique to Colombia, as it was not found in sequences reported thus far from Venezuela or Ecuador. This regional specificity has been observed for other minor lineages, such as C.1.1 currently circulating in Brazil, while its sibling, C.1.2, has been found in the Dominican Republic and Haiti [32]. The other subclade grouped sequences from Ecuador together with four Colombian sequences (PUT/17783320/2013; PUT/17783321/2013; CAL/17783325/2013; CAU/17783319/2013), it was separated 0.0056 substitutions per site, corresponding to 57 inferred substitutions, therefore was denominated as D.3.2 *putative* (Fig 1b). For minor lineage D.2, analysis revealed diversification into two subclades, designated D.2.1 and D.2.2 putative. D.2.1 comprises Colombian sequences mainly and exhibited 60 inferred substitutions along the ancestral branch. Subclade D.2.2 comprising sequences collected in northern and southwestern Colombia, Venezuela, and Ecuador (the latter of which are exclusively available in the GISAID database). It was separated from the common ancestor by 0.0038 substitutions per site, corresponding to 39 inferred substitutions (Fig 1b, S3 Table).

### Spatio-temporal analysis of genotype III in Colombia

We performed a root-to-tip regression analysis using TempEst on a dataset of 161 sequences. The analysis revealed a strong temporal signal, with a positive linear correlation (S1 Fig). The residual mean squared (RMS) value was 4.66 × 10 ⁻ ⁶, indicating a good fit to the molecular clock model. These results suggest highly clock-like evolution in the dataset, supporting its suitability for time-calibrated phylogenetic analyses. No outlier sequences were identified or removed. The estimated substitution rate, derived from the slope of the regression line, was approximately 9.05 × 10 ⁻ ⁴ substitutions/site/year, consistent with previously reported rates (7.37 × 10^−4^, 9.5 × 10^−4^ subs./site/year) for DENV-2 [33]).

Model comparison based on marginal likelihood estimates using path sampling favored the relaxed molecular clock model over the strict clock model (BF = 331.7), therefore, subsequent phylogenetic inferences were based on the relaxed clock model. The maximum clade credibility (MCC) tree obtained under this model revealed a well-resolved phylogeny. Most nodes were strongly supported, with posterior probabilities >0.9. The estimated time to the most recent common ancestor (TMRCA) for the entire dataset was 1981 (95% HPD 1978–1984).

**Fig 2:**
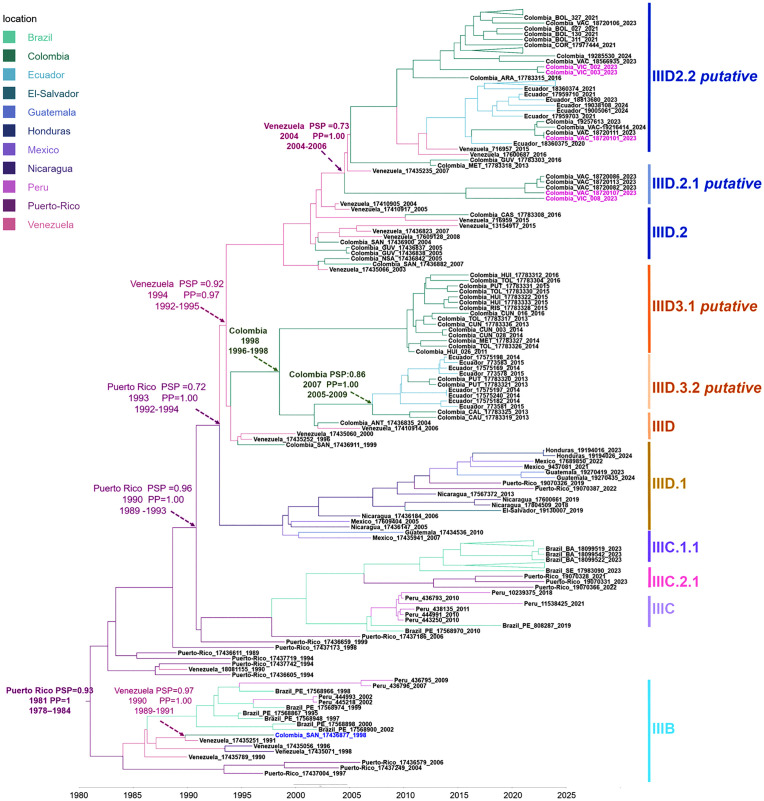
Spatiotemporal analysis of DENV-2 genotype III in American countries based on complete ORF sequences. The maximum clade credibility tree was inferred using 161 sequences (10,170 nt). The time to the most recent common ancestor (TMRCA) was estimated using the year of isolation as the calibration point, under a relaxed log normal molecular clock, with the GTR model and discrete gamma distribution. The estimated years of TMRCA existence are shown for key nodes. PSP: posterior state probability. [HPD]: 95% highest posterior density. PP: posterior probability.

Our analysis confirmed previous studies reporting the introduction of lineage B to the Americas around 1981 via Puerto Rico [33], and its introduction into Colombia from Venezuela around 1990 (PSP = 0.98; PP = 1.00). The only Colombian sequence reported in 1998 from the Santander province suggested that, as in other countries, lineage 2III_B was subsequently replaced (Fig 2). Although lineages C and D share a common origin in Puerto Rico around 1990 (PSP = 0.96; PP = 1.00), phylogeographic analysis confirmed previous studies showing that lineage C spread to South America via Brazil and then Peru, while lineage D has spread via Venezuela, followed Colombia and then Ecuador. Despite the close geographical proximity of Colombia and Peru, no evidence of dissemination between the two countries has been observed using this dataset. Conversely, a continuous ‘exchange’ can be noted, mainly between Venezuela-Colombia and Colombia-Ecuador. Although complete genomes from Ecuador are only available from 2014 onwards, our analysis suggests that Colombia is the most likely origin of the minor lineage D.3 (PSP = 0.86; 2007.6; PP = 1.0). For minor lineage D.2, Venezuela might be the origin. However, there is no strong evidence to support this (PSP = 0.50; 2012; PP = 0.99) (Fig 2).

**Fig 3:**
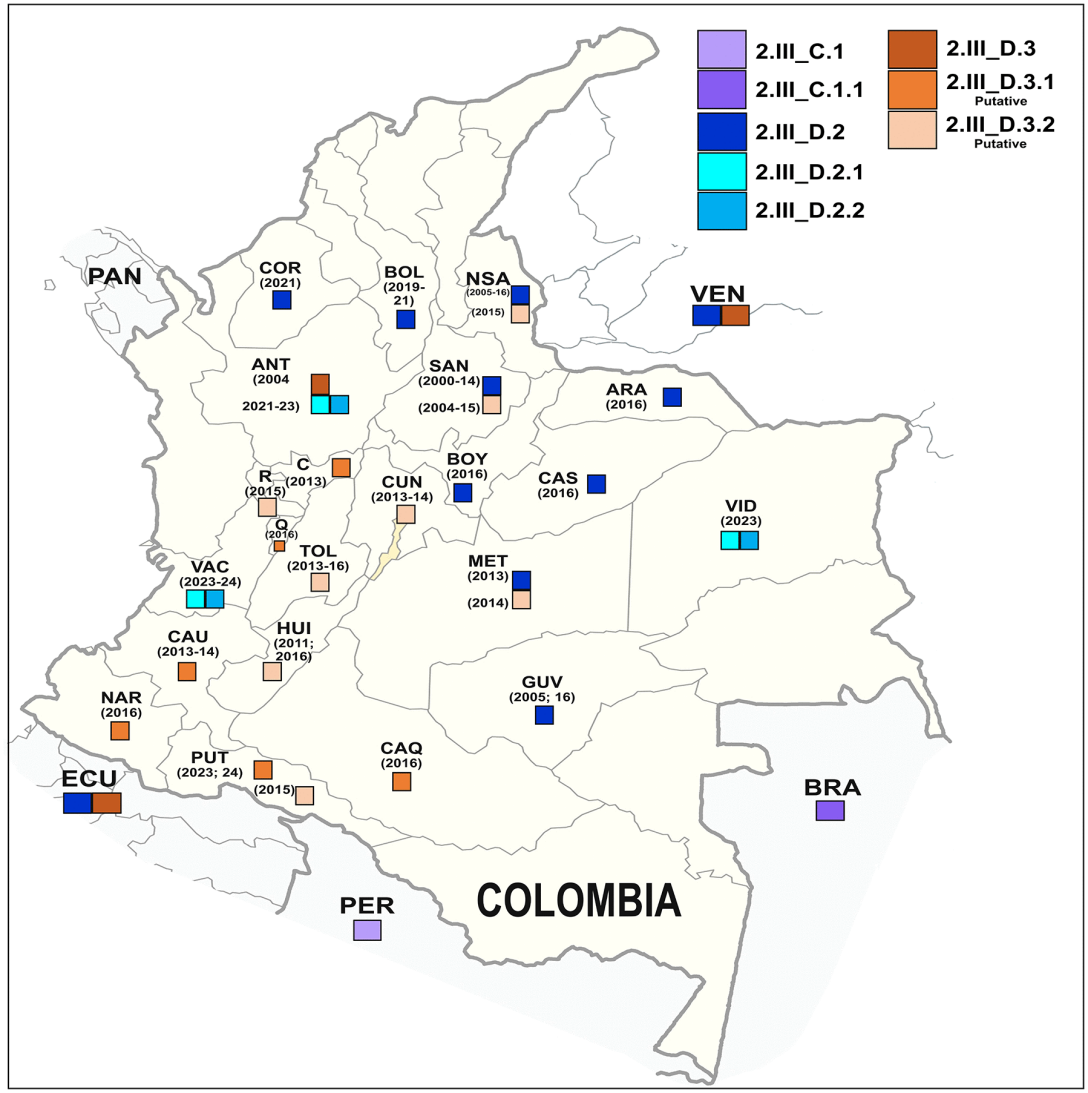
Differential geographical distribution of the minor lineages D.2 and D.3 in Colombia. Abbreviations used: Córdoba COR; Bolívar BOL; North of Santander NSA; Santander SAN; Arauca ARA; Casanare CAS; Meta MET; Vichada VID; Putumayo PUT; Valle del Cauca: VAC; Huila HUI; Tolima TOL; Risaralda RIS; Cundinamarca CUN. Base layer map for Colombia and neighborhood countries was obtained from open files of https://www.mapchart.net/americas-detailed.html. We are allowed to use, edit and modify this map created with MapChart, under a Creative Commons Attribution 4.0 International license (CC BY 4.0).

In Colombia, two separate introductions likely from Venezuela gave rise to distinct clades corresponding to the minor lineages D.3 and D.2 (Fig 2). The D.3 lineage diverged around 1994 (95% HPD 1993–1994; PP = 0.99), subsequently dividing into two subclades with a TMRCA around 1998 (95% HPD: 1996–1998; PP = 1.00). One of these subclades, D.3.1, comprises viruses that circulated in departments located in the Colombian Andean zone, such as Huila, Tolima, Risaralda and Cundinamarca, between 2011 and 2016. The other subclade comprises mainly Ecuadorian sequences collected between 2014 and 2015 (Figs 2 and 3).

The minor lineage D.2 is geographically widespread, extending from Santander and Orinoquía (provinces of Arauca, Casanare, Meta, and Vichada) to Bolívar and Córdoba in the north, and extending to the province of Valle del Cauca in the south-west (Fig 3). Notably, the sequences reported in the last five years fall into two subclades D.2.1 and D.2.2, which diverged almost 20 years ago (2004 PP = 1.00, 95% HPD: 2004–2006) (Fig 2). Despite this, the two minor lineages currently circulate in the same provinces, for example, sequences VIC/002/2023 and VIC/003/2023, which belong to the minor lineage D.2.1, were collected in the same municipality in the Vichada province as sequence VIC/008/2023, which belongs to the minor lineage D.2.2. Sequences VAC/18720101/2023 (D.2.1) and VAC/18720107/2023 (D.2.2) were collected in the same week, but in two cities only 29 km apart. Unfortunately, the lack of complete genomes from other provinces limits our ability to determine whether these two minor lineages are co-circulating in other regions of the country.

Because more E-gene sequences than complete genomes were available, we analyzed all E sequences from Colombia deposited in the NCBI database up to December 2024 (S4 Table). These results indicate that the minor lineages D.2.1 and D.2.2 have co-circulated in the department of Antioquia since 2021 (S4 Table). No additional sequence data were identified for other provinces. This finding underscores the need for more comprehensive genomic surveillance across Colombia to clarify the spatial distribution and dynamics of these DENV2 III minor lineages.

### Amino acid mutation analysis for the minor lineages D.2 and D.3

The mutation profiles of the minor lineages found in Colombian sequences are clearly distinguishable, indicating significant genetic differentiation among them. The minor lineage D.3 is distinguished from D.2 by changes in four amino acids: E-742, NS1–868, NS2A-1236 and NS5–2866 (Table 2). The minor lineages D.3.1 and D.3.2 differ in 19 amino acids, thirteen changes are exclusive for D.3.1 and four for D.3.2. The changes were primarily found in the non-structural proteins. NS5 has the highest number of differences with five (T2496I, T2510A, K3140R, E3298D, N3367T), followed by NS4, NS3 and NS2A with three mutations each.

**Table 2:** Distinctive minor lineage mutations.

PROTEIN	Position in the Polyprotein	D3	D.3.1	D.3.2	D.2	D.2.1	D.2.2
**CAPSIDE 1–114**	**17**	**K**	K	K	K	**K**	**R**
	**26**	**V**	**M**	V	V	V	V
**ENVELOPE 281–776**	**450**	**I**	I	T	I	**I**	**T**
	**604**	**V**	V	V	V	I	I
	**639**	**T**	T	T	I	I	T
	**641**	**K**	K	K	K	K	**R**
	**742**	**I**	I	I	V	V	V
**NS1 777–1128**	**858**	**E**	E	E	E	**D**	E
	**868**	**I**	I	I	T	T	T
	**873**	**Q**	Q	Q	Q	**P**	Q
	**945**	**K**	K	K	K	**R**	K
	**949**	**K**	**R**	K	K	K	K
	**952**	**V**	V	V	V	V	**A**
	**953**	**F**	**V**	F	F	F	F
	**1054**	**F**	F	F	F	F	F
**NS2A 1129–1346**	**1129**	**H**	H	H	H	**H**	**L**
	**1236**	**A**	A	A	T	T	T
	**1242**	**T**	**A**	T	T	T	T
	**1266**	**N**	**S**	N	N	N	N
	**1289**	**Q**	Q	Q	R	Q	R
	**1301**	**A**	A	A	A	**T**	A
	**1316**	**A**	**S**	A	A	A	A
**NS2B 1347–1477**	**1356**	**I**	I	**V**	I	I	I
	**1385**	**V**	V	V	V	**I**	V
	**1402**	**A**	A	A	A	**T**	A
	**1432**	**K**	K	K	K	**R**	K
**NS3 1478–2096**	**1489**	**E**	G	E	G	**E**	G
	**1590**	**I**	**T**	I	I	I	I
	**1616**	**D**	D	**N**	D	D	D
**NS4 2097–2240**	**2105**	**R**	**K**	R	R	R	R
	**2152**	**T**	**A**	T	T	T	T
	**2186**	**I**	**I**	V	V	V	V
**NS4B 2241–2490**	**2266**	**S**	S	S	S	S	**P**
	**2267**	**E**	E	E	E	E	**D**
	**2380**	**A**	A	A	A	A	**T**
**NS5 2491–3391**	**2496**	**I**	**T**	I	I	I	I
	**2510**	**A**	**T**	A	A	A	A
	**2866**	**K**	K	K	R	R	R
	**2879**	**K**	K	K	K	**E**	K
	**2920**	**G**	S	G	G	G	G
	**2928**	**R**	R	R	R	K	K
	**3014**	**G**	G	G	G	G	G
	**3020**	**A**	V	V	V	V	V
	**3139**	**A**	A	A	A	**T**	A
	**3140**	**R**	**K**	R	R	R	R
	**3178**	**V**	V	V	V	V	**I**
						
	**3298**	**E**	E	**D**	E	E	E
	**3367**	**N**	N	**T**	N	N	N

All available Colombian sequences for major lineage D were compared at the amino acid level with SAN/17436911/1999, the oldest reported sequence for this lineage. Mutations specific to each minor lineage are highlighted. Those that differentiate D.2 from D.3 are highlighted in gray.

Only two variations were identified in structural proteins: one in C (V26M), and one in E (T450I). Minor lineages D.2.1 and D.2.2 differ in 20 amino acids, with nine unique to D.2.1 and eight to D.2.2. Four changes were found in structural proteins and four in NS1. The NS2A, NS2B, NS4B AND NS5 proteins differ by three amino acids each (Table 2). Some of these mutations may be associated with virulence, pathogenicity, and/or vector competence, functional studies will be required to determine their actual impact on the phenotype and/or clinical presentation of the disease.

### Selection pressure analysis

To study the selection pressure exerted on DENV-2 genotype III in Colombia, the dataset for the Colombian ORFs (n = 59, 3,390 sites) was analyzed using MEME, FEL, SLAC, and FUBAR methods. A total of 260 codons found to be under negative selection were identified by three methods, while only two sites were detected to be under positive selection pressure (Table 3). Positive selection was considered if the four methods showed nonsynonymous (β)/ synonymous (α) substitution ratio > 1. These sites were codon 1289 in the NS2A gene and codon 1662 in the NS3 gene. Q1289 was observed in strains belonging to the D.3 minor lineage, while R1289 was present in D.2 sequences (Table 2). The R1662K mutation was detected in 4 out of 59 sequences but was not associated with a particular lineage.

**Table 3:** Codons under selection pressure.

		SLAC			FEL			FUBAR			MEME		
Negative Selection	Total	275			537			1060			N/A		
**Positive Selection**	**Codon**	**α**	**β**	**p**	**α**	**β**	**p**	**α**	**β**	**PP**	**α**	**β**	**p**
	1289	0.00	5.00	0.050	0.00	8.97	0.013	0.83	26.40	0.95	0.00	0.00	0.02
	1662	0.00	10.00	0.023	0.00	7.49	0.018	0.84	20.11		0.00	0.00	0.03

The codons under significant evidence of positive selection (p < 0.1) identified by four methods are shown. Synonymous (α) and non-synonymous (β) substitution rates over sites.

PP: posterior probability.

## Discussion

Colombia is one of the most severely affected countries by dengue in the Americas, with a dramatic increase in case numbers in recent years. The last major outbreaks were reported in 2019 with 124,989 cases and most double the number of cases in 2024 [34]. Since 2000, when the national surveillance system began recording DENV serotypes, all four serotypes have been documented, with DENV-1 and DENV-2 showing the highest circulation rates [35]. Although DENV-1 was unquestionably the serotype causing the 2019 epidemic, DENV-2 has been continuously circulating in several regions of the country over the past 20 years [35]. In this study, we investigated the evolutionary and phylogeographic patterns of DENV-2 using novel sequences as well as sequences reported in databases up to January 2025. We confirmed the exclusive circulation of genotype III up to 2023, which is consistent with the results of other studies [14,15,36].

The evolutionary analysis showed four major clades corresponding to the major lineages identified by the Genome Detective tool for this genotype. DENV-2III_B likely arose in Puerto Rico by 1981, was disseminated to the Caribbean and then arrived in South America by 1990 and appeared to become extinct around 2009. Lineage C in Greater Antilles, Brazil and Peru, and Lineage D in Central America, Venezuela, Colombia, and Ecuador have been circulating for the past 20 years and have been responsible for several outbreaks [33].

In Colombia, we identified two minor lineages: D.3. which circulated between 2011–2016 in the Andean region, and D.2, with a broader temporal (2004–2024) and spatial circulation (Figs 2-3). These two minor lineages correspond to lineages 1 and 2 previously described by Laiton-Donato et al. in 2019 [15]. Their study of 48 Colombian E-gene sequences proposed a differential geographical distribution and the presence of non-synonymous substitutions accompanying lineage diversification, although only one substitution (I462V) was associated with the emergence of lineage 2. Our analysis of the entire open reading frame revealed greater diversification of lineage 1 into the minor lineages D.3.1 and D.3.2, as well as of lineage 2 into the minor lineages D.2.1 and D.2.2 (Fig 1). It also revealed the presence of distinctive mutations for each lineage (Table 2).

In countries such as Puerto Rico, Peru, Nicaragua, and Brazil [33,37], the introduction of new DENV-2 lineages has been associated with the displacement of older lineages. However, in Colombia, it is not possible to establish this definitively, given the differential geographical distribution of minor lineages and the lack of recent information for some provinces. Two scenarios are therefore possible: The two dominant lineages could have remained in circulation in different geographical regions, or minor lineage D.3 could have been displaced by D.2. This alternative is plausible, given that our analysis of all the available Colombian E-gene sequences in the NCBI database confirmed that the minor linage D.2 has circulated in Colombia from 1998 to the present day, whereas minor linage D.3 was detected in sequences that circulated before 2016.

A recent study by Martínez et al. [38] described the circulation of two new lineages in Colombia. They refer to A and B lineages for DENV-2 genotype III rather than the major or minor lineages proposed by Hill et al. [32]. Unfortunately, the Colombian sequences for Martínez’s study have not yet been published, so they could not be analyzed using the parameters proposed by Hill et al. [32]. However, as the analyzed sequences were collected in recent years, they most likely belong to the minor lineage D.2. The “A and B lineages” may coincide with the minor lineages D.2.1 and D.2.2 described here, and by Grubaugh et al. [39] in viruses collected between 2023 and 2024. Similar to our findings, Grubaugh’s study identified D.2 sequences that had diverged into two clades around 2004 [39].

The phylogeographic analysis corroborated that Venezuela serves as a major hub for the spread of this genotype to Colombia, as previously described [33]. A prior study, conducted with 406 E-gene sequences from 29 countries in the Americas over a 30-year period, revealed the existence of two main routes for the spread of this genotype from the Caribbean to South America, separated by the Andes Mountains. The western route, after reaching Venezuela from the Antilles, continued through Colombia and Ecuador, while the eastern route proceeded through Brazil, Bolivia, and Paraguay [33]. Although the authors suggested that both routes converge in Peru, our full genome analyses found no evidence of such dispersal from the western route. Our results are consistent with those of Maljkovic [40], who also failed to identify a dissemination route from Peru to Ecuador when analyzing a set of 314 genomes.

Our analysis identified two minor lineages in Ecuador: D.3 in strains that circulating between 2014–2015 and D.2 in strains circulating between 2020–2024. These findings are consistent with the two clades reported by Márquez et al. in viruses circulating in northwestern Ecuador [41]. Although Maljkovic et al. identified Venezuela as the country of origin of the viruses reported in 2014–2015 [40], it should be noted that their dataset did not include the 20 Colombian genomes that gave rise to minor lineage D.3.

Finally, to study the selection pressure exerted on genotype III in Colombia, we analyzed the Colombian ORFs. Limited evidence of positive selection was identified, suggesting that the viruses were under strong purifying selection, as previously shown for this genotype in Central American strains [42]. In our study, positive selection was significant in only two codons, NS2A-1289 and NS3–1662. No codon under positive pressure was detected in the E protein, in contrast with previous reports for American strains from Nicaragua and Puerto Rico [37,42]. Positive selection in nonstructural genes has been reported for this genotype earlier, Añez et al. [42] identified codons NS2A-2, NS3–180, NS3–437, NS5–269, NS5–523, and NS5–850. Another study found that codons NS5–558 and NS4B-156 distinguish Asian strains from all American strains [43]. The NS5–811 codon was identified in the lineage causing the 2010–2011 outbreak in Peru, and mutations A811V and I277V were detected in circulating strains [44]. Nonstructural proteins participate in genome replication, virion assembly [45], and antiviral response [46]. Positive selection on NS proteins might optimize viral fitness by modulating replication efficiency and/or favoring evasion of the host immune system.

While positive selection could explain the differential success among lineages, we did not find any association between positively selected codons and clade emergence. Therefore, the movement of viral strains caused by human flows between regions due to globalization, trade, tourism, and migration, as well as seasonal variations and the wide distribution of vectors in Colombia, may provide new routes of entry and increase the likelihood of the emergence of new variants.

These findings highlight the complex evolutionary dynamics of DENV-2 genotype III in Colombia, with distinct mutation patterns characterizing major and minor lineages. The identification of sites under positive selection pressure suggests potential adaptive changes in the virus, which may have implications for its fitness, transmission, or interaction with the host immune system. Further functional studies are needed to elucidate the biological significance of these mutations and their potential impact on viral phenotype and epidemiology.

## Conclusion

This study provides a comprehensive analysis of the spatial and temporal dynamics of DENV-2 genotype III in Colombia (1998–2024) using whole-coding genome sequences. Our results reveal frequent introductions of new viral strains—predominantly linked to Venezuela—and show how these introductions have fostered co-circulation and diversification of DENV lineages within Colombia. Notably, we document the current co-circulation of two distinct minor lineages, D.2.1 and D.2.2, which appear to have evolved independently from the D.2 minor lineage. Importantly, we demonstrate the practical utility of the recently proposed hierarchical lineage nomenclature for DENV, which facilitates standardized reporting and cross-country comparisons and thus strengthens regional genomic surveillance and the prioritization of lineages for epidemiological and functional follow-up.

## Supporting information

S1 Table Primers used to amplify the full genome DENV2 sequence. (XLSX)

S2 Table Characteristics of the novel sequences reported in this study. (XLSX)

S3 Table GenBank and GISAID accession numbers of sequences used to define new minor lineages. (XLSX)

S4 Table Analysis of DENV-2 III E-gene sequences reported between 1989 and 2024 using the Genome Detective Tool. (XLSX)

S1 Fig Root to trip regression analysis. Plot of the root-to-tip genetic distance against sampling time are shown for ML phylogeny estimated from 161 sequences, sampled between 1987 and 2024. The correlation coefficient was estimated by TempEst.
(TIF)
